# 3-Axis Fully-Integrated Capacitive Tactile Sensor with Flip-Bonded CMOS on LTCC Interposer †

**DOI:** 10.3390/s17112451

**Published:** 2017-10-25

**Authors:** Sho Asano, Masanori Muroyama, Takahiro Nakayama, Yoshiyuki Hata, Yutaka Nonomura, Shuji Tanaka

**Affiliations:** 1 Department of Robotics, Graduate School of Engineering, Tohoku University, 6-6-01 Aramaki Aza Aoba, Aoba-ku, Sendai, Miyagi 980-8579, Japan; tanaka@mems.mech.tohoku.ac.jp; 2 Micro System Integration Center, Tohoku University, 519-1176 Aramaki Aza Aoba, Aoba-ku, Sendai, Miyagi 980-0845, Japan; muroyama@mems.mech.tohoku.ac.jp; 3 Partner Robot Division, Toyota Motor Corporation, 543 Kirigahora, Nishi-hirose-cho, Toyota, Aichi 470-0309, Japan; takahiro_nakayama_aa@mail.toyota.co.jp; 4 System & Electronics Engineering Department III, Toyota Central R&D Labs., Inc., 41-1 Yokomichi, Nagakute, Aichi 480-1192, Japan; yhata@mosk.tytlabs.co.jp (Y.H.); nonomura@meijo-u.ac.jp (Y.N.)

**Keywords:** 3-axis tactile sensor, MEMS-CMOS integration, capacitive sensor, Au-Au thermo-compression bonding, low temperature cofired ceramic

## Abstract

This paper reports a 3-axis fully integrated differential capacitive tactile sensor surface-mountable on a bus line. The sensor integrates a flip-bonded complementary metal-oxide semiconductor (CMOS) with capacitive sensing circuits on a low temperature cofired ceramic (LTCC) interposer with Au through vias by Au-Au thermo-compression bonding. The CMOS circuit and bonding pads on the sensor backside were electrically connected through Au bumps and the LTCC interposer, and the differential capacitive gap was formed by an Au sealing frame. A diaphragm for sensing 3-axis force was formed in the CMOS substrate. The dimensions of the completed sensor are 2.5 mm in width, 2.5 mm in length, and 0.66 mm in thickness. The fabricated sensor output coded 3-axis capacitive sensing data according to applied 3-axis force by three-dimensional (3D)-printed pins. The measured sensitivity was as high as over 34 Count/mN for normal force and 14 to 15 Count/mN for shear force with small noise, which corresponds to less than 1 mN. The hysteresis and the average cross-sensitivity were also found to be less than 2% full scale and 11%, respectively.

## 1. Introduction

Service robots such as nursing care robots, home assistant robots, and entertainment robots are now being developed all over the world. In contrast with industrial robots, they operate in human environments like homes and hospitals without fences and other safety measures. Tactile information about physical contact allows for dexterous manipulation and safe operation in direct human-robot interaction applications. For these purposes, whole-body tactile sensing is highly desired. 3-axis force (normal and shear force) sensing is also important to detect the direction of the contact force and slip of grasping objects [1,2].

As for whole-body tactile sensing, some papers have reported distributed tactile sensors on a large area of a humanoid robot [3,4,5]. In order to reduce the number of wires, serial bus connection [3,4], or matrix scanning [5] are used. Flexible printed circuit boards (PCBs) also allow for conformable implementation on curved surfaces of a robot. However, the discrete components on PCBs are relatively large in size, which causes low spatial resolution. Recently, 3-axis tactile sensors have been developed based on microelectromechanical systems (MEMS) technology [6,7]. In [8], a piezoresisitive tactile sensor with surface-doped and sidewall-doped beams embedded in polydimethylsiloxane (PDMS) was presented. This sensor demonstrated measurement of 3-axis components of applied pressure independently. In [9], the slip detection of a grasping object was demonstrated by microcantilevers with a thin film strain gauge embedded in PDMS. In these studies, the tactile sensor and the readout circuit are connected one-by-one, therefore the number of wires will increase for connecting many sensors. Flexible tactile sensor arrays based on strain gauges [10] and multiple capacitors [11] demonstrated high spatial resolution like human fingertips, but the wiring problem was not completely solved. Another problem is long wire connection between the tactile sensor and the readout circuit, which increases noise and parasitic capacitance. The monolithic integration of MEMS sensor and complementary metal-oxide semiconductor (CMOS) circuit (MEMS-CMOS integration) enables the reduction of noise and parasitic capacitance, as well as the miniaturization of the device. CMOS-integrated tactile sensor arrays with a spatial resolution of 1 mm or higher have been reported [12,13]. Also, a capacitive tactile sensor implemented by CMOS standard process and in-house post-CMOS process has been proposed [14]. However, detection of shear force is not examined by these CMOS-integrated tactile sensors.

We have developed a tactile sensor, which can be surface-mounted on a flexible and stretchable bus line by integrating a MEMS capacitive sensor and a CMOS circuit [15]. The integrated tactile sensor can detect contact force by a diaphragm with a boss formed in the CMOS substrate. Thermo-compression bonding by planarized Au bumps and an Au sealing frame is used to integrate the CMOS on an low temperature cofired ceramic (LTCC) interposer, which provides interconnections from the CMOS circuit to the bus lines. The sensor has demonstrated the following functions: (1) data reduction by human-inspired threshold and adaptation operations; and (2) the adjustment of sensitivity and data rate after implementation. However, this integrated tactile sensor can detect normal force only because of limited functionalities.

**Figure 1:**
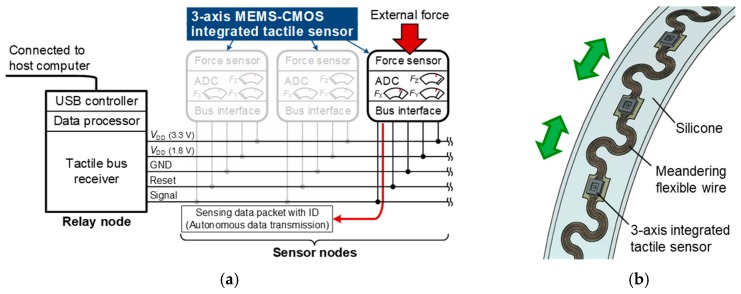
Conceptual figure of the 3-axis integrated tactile sensors on a flexible and stretchable bus line: (**a**) System diagram; (**b**) Physical implementation.

The objective of this study is to develop a CMOS-integrated 3-axis tactile sensor surface-mountable on a bus line. Our final target is a networked event-driven tactile sensor system with numerous distributed 3-axis integrated tactile sensors on a flexible and stretchable bus line, as shown in Figure 1. In this study, we focus on the 3-axis integrated tactile sensor. In order to detect 3-axis force, we have used a new CMOS substrate with four channels of differential capacitive sensing circuits. Also, we have improved the sensor structure from the previous study to enhance sensitivity to not only normal force but also shear force [16]. This paper is organized as follows. In Section 2, we show the structure and working principle of the sensor and the results of finite element method (FEM) simulation. In Section 3, the fabrication process of the sensor is presented. In Section 4, an experimental setup for evaluation and the measurement results of normal force and shear force are described. Finally, Section 5 concludes this paper.

## 2. Design

### 2.1. Sensor Structure

**Figure 2:**
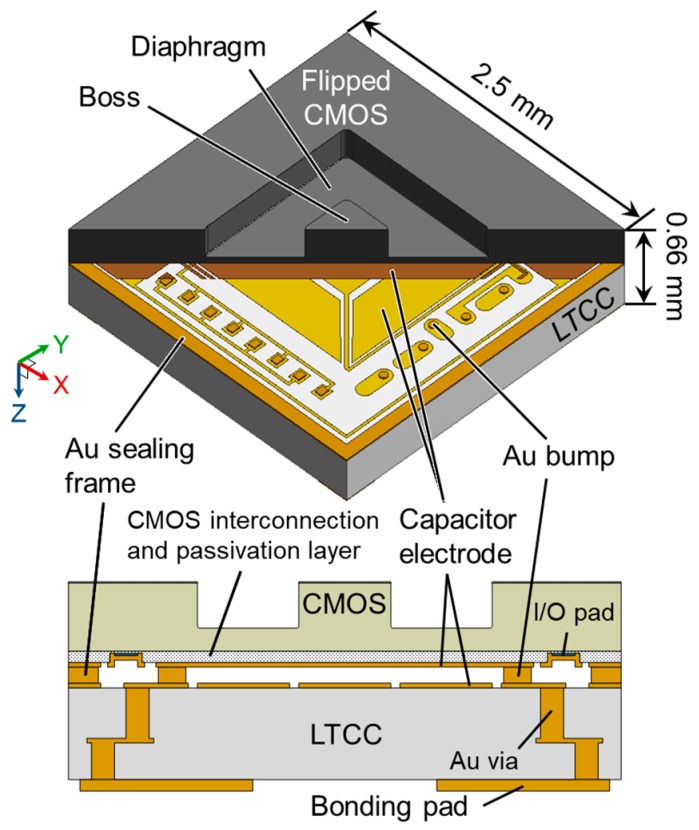
Structure of the 3-axis integrated tactile sensor.

Figure 2 shows the structure of the 3-axis integrated tactile sensor. The dimensions of the sensor are designed to be 2.5 mm in width, 2.5 mm in length, and 0.66 mm in thickness. The sensor has a flipped CMOS with a sensing diaphragm above an LTCC interposer. The CMOS circuit is used for differential capacitive sensing and serial bus communication. Interconnections from the CMOS circuit are provided through Au bumps and Au vias of the LTCC interposer, which makes the fabrication process simpler as compared to using through silicon vias (TSVs) [17,18] or through silicon grooves [19]. The LTCC interposer is suitable for wafer-level packaging due to three-dimensional (3D) internal wiring as well as its small mismatch of coefficient of thermal expansion compared to Si [20]. The capacitive sensor structure is sealed with an Au sealing frame. Although we have formed a diaphragm with tapered boss by anisotropic wet etching in the previous study [15], the side surface of the diaphragm and the boss is vertical in this study to enhance sensitivity to normal force and shear force. The capacitive gap of 4.5 µm, which is smaller than that of the previous sensor (10 µm), also enables the enhancement of sensitivity and the prevention of large diaphragm deflection. As described later, differential capacitive sensing like a seesaw for *X*-Axis and *Y*-Axis allows shear force detection with high sensitivity while one capacitance changes and another is fixed for *Z*-Axis.

### 2.2. Working Principle

**Figure 3:**
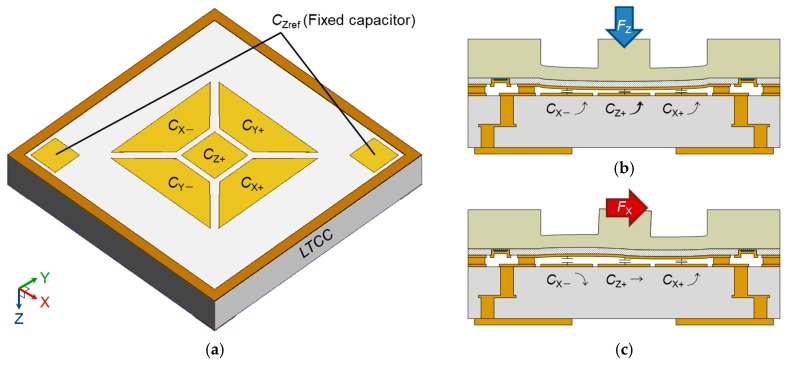
(**a**) Electrode layout; Working principles for (**b**) normal force *F*_Z_ and (**c**) *X*-Axis shear force *F*_X_.

Figure 3a shows the electrode layout of the 3-axis integrated tactile sensor. The sensor has 5 sensor capacitors *C*_X−_, *C*_X+_, *C*_Y−_, *C*_Y+_, and *C*_Z+_ under the diaphragm, and a fixed capacitor *C*_Zref_, which is apart from the other electrodes. When normal force *F*_Z_ is applied to the boss of the sensor, capacitances except for *C*_Zref_ increase as shown in Figure 3b. In the case that shear force is applied, capacitances correspond to the force direction change. For example, if *X*-Axis shear force *F*_X_ is applied to the sensor in positive direction as shown in Figure 3c, *C*_X+_ increases while *C*_X−_ decreases. The other capacitances do not change in this case.

**Figure 4:**
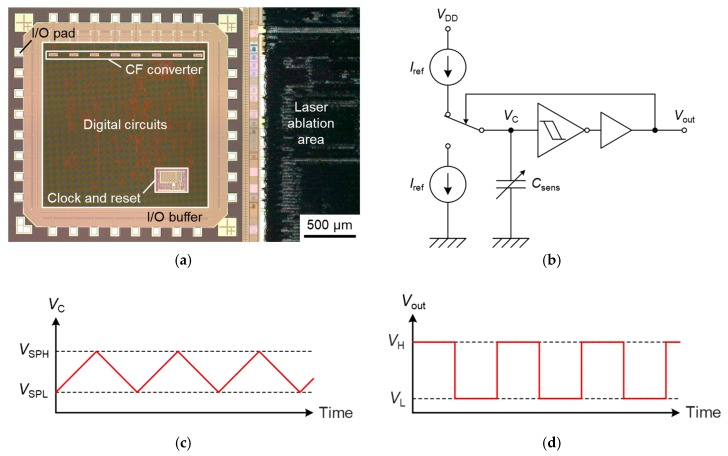
(**a**) Optical micrograph of the complementary metal-oxide semiconductor (CMOS) substrate; (**b**) Schematic diagram of the capacitive readout circuit; Time responses of (**c**) the voltage of the sensor capacitor *V*_C_ and (**d**) the oscillation output voltage *V*_out_.

The capacitance change is detected by the CMOS circuit. Figure 4 shows the optical micrograph of the CMOS substrate and the schematic diagram of the readout circuit. The CMOS circuit has a capacitance-frequency (CF) converter for each capacitor, and the oscillation frequency *f* is written as

(1) $$f=\frac{4.74\times 10^{4}}{C_{\mathrm{sens}}+497},$$

where *C*_sens_ is the sensor capacitance and the units of *f* and *C*_sens_ are MHz and fF, respectively. The 3-axis output values *N*_X_, *N*_Y_, and *N*_Z_ are given by

(2) $$N_{X}=f_{X-}\cdot \frac{P}{f_{\mathrm{clock}}}-f_{X+}\cdot \frac{P}{f_{\mathrm{clock}}},$$

(3) $$N_{Y}=f_{Y-}\cdot \frac{P}{f_{\mathrm{clock}}}-f_{Y+}\cdot \frac{P}{f_{\mathrm{clock}}},$$

(4) $$N_{Z}=f_{\mathrm{Zref}}\cdot \frac{P}{f_{\mathrm{clock}}}-f_{Z+}\cdot \frac{P}{f_{\mathrm{clock}}},$$

where the subscripts of *f* correspond to the capacitor (e.g., *f*_Zref_ is the oscillation frequency of *C*_Zref_), and *P* and *f*_clock_ are the count period and the clock frequency, respectively. In this study, *P* and *f*_clock_ are set to be 2^13^ clock cycles and 1.56 MHz, respectively. When *F*_Z_ is applied, *N*_Z_ increases, because *f*_Zref_ is fixed and *f*_Z+_ decreases. In contrast, *N*_X_ and *N*_Y_ do not change because the decreases of *f*_X−_ and *f*_X+_ as well as *f*_Y−_ and *f*_Y+_ are the same in principle due to the symmetry of the electrode layout. If *F*_X_ is applied to the positive direction, *N*_X_ increases because *f*_X−_ increases and *f*_X+_ decreases, while *N*_Y_ and *N*_Z_ do not change because the other oscillation frequencies do not change.

### 2.3. FEM Simulation

FEM simulation software (Femtet, Murata Software Co., Ltd., Tokyo, Japan) was used to estimate the diaphragm deflection and the capacitance change when force is applied to the 3-axis integrated tactile sensor. The 3-axis integrated tactile sensor is designed to distribute on the bus lines, and the contact force is divided to some sensors. Thus, the maximum applied force was set to 1 N on the assumption of normal manipulation operations. The target minimum detectable force was decided as 1 mN for achieving a dynamic range of 1000:1. The measurement range can be changed by the dimensions of the sensor (e.g., the diaphragm thickness, the size of the boss, and the thickness of the Au bumps, and the sealing frame). The dimension of the diaphragm was determined to be 1.3 mm square, in which no analog circuits is located, to avoid piezoelectric effect to the analog circuits. The thickness of the diaphragm was determined to be 50 µm, as with the previous study [15]. The dimension of the boss is 0.4 mm square. In this simulation as well as the measurement described in Section 4, normal force was applied to the top surface of the boss, and shear force was applied to one of the side surfaces from the top to 200 µm below the top of the boss.

**Figure 5:**
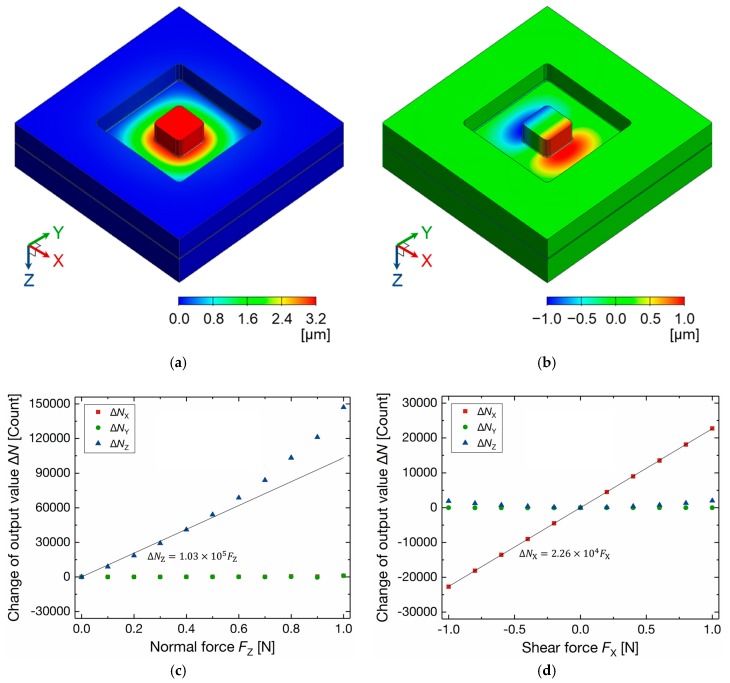
Finite element method (FEM) simulation results: Diaphragm deflection by (**a**) normal force *F*_Z_ of 1 N and (**b**) *X*-Axis shear force *F*_X_ of 1 N applied to the positive direction; Calculated change of 3-axis output values by (**c**) *F*_Z_ and (**d**) *F*_X_.

Figure 5a,b show the deflection of the diaphragm by normal force *F*_Z_ and *X*-Axis shear force *F*_X_ of 1 N applied to the positive direction, respectively. The maximum deflections of 3.21 µm for *F*_Z_ and 1.03 µm for *F*_X_ are smaller than the initial capacitive gap of 4.5 µm. The *Z*-Axis stiffness was calculated as 3.1 × 10^5^ N/m. The maximum principal stresses for *F*_Z_ and *F*_X_ of 1 N were estimated as 365 MPa and 542 MPa, respectively. They are smaller than the reported fracture stress of Si specimens microfabricated by deep reactive ion etching (DRIE) [21]. The eigenfrequencies of the parallel motion of the diaphragm and the rotation motion of the boss were estimated to be 223 kHz and 399 kHz, respectively. These values are much higher than the frequency of the motion of robots and the detectable stimuli frequency of human tactile receptors. Figure 5c,d show the change of 3-axis output values calculated by the simulated 3-axis capacitances as a function of *F*_Z_ and *F*_X_, respectively. The nonlinearity of the change of *N*_Z_ is caused by the relationship of the oscillation frequency and the capacitance, as described in Equation (1). The sensitivity was estimated as 103 Count/mN for *F*_Z_ from 0 N to 0.5 N and 22.6 Count/mN for *F*_X_ from −1 N to 1 N, which is high enough to satisfy the minimum force sensitivity of 1 mN. The capacitance at unload condition was calculated as 643 fF for *C*_X−_, *C*_X+_, *C*_Y−_, and *C*_Y+_, 368 fF for *C*_Zref_, and 332 fF for *C*_Z+_.

## 3. Fabrication

**Figure 6:**
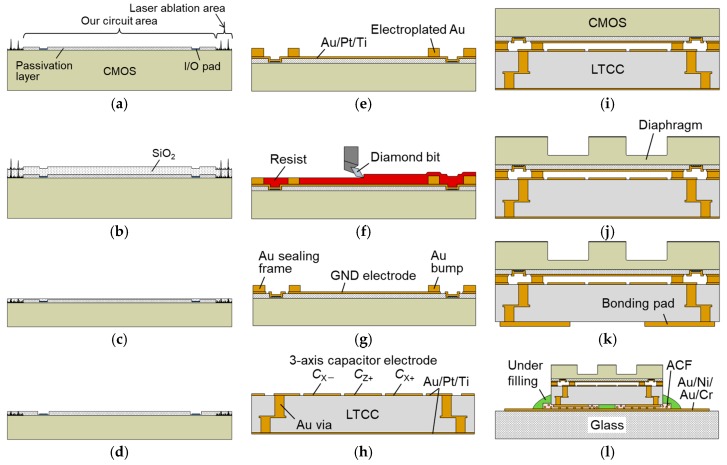
Fabrication process of the 3-axis integrated tactile sensor: (**a**) Received CMOS substrate; (**b**) Thick SiO_2_ film deposition; (**c**) Planarization and back grinding; (**d**) Pad opening; (**e**) Au electroplating; (**f**) Planarization of Au bumps and an Au sealing frame; (**g**) Formation of a ground (GND) electrode and rewiring; (**h**) Formation of 3-axis capacitor electrodes and rewiring; (**i**) Au-Au thermo-compression bonding; (**j**) Formation of a diaphragm; (**k**) Formation of bonding pads; (**l**) Surface mounting on a glass substrate with interconnections by an anisotropic conductive film (ACF).

**Figure 7:**
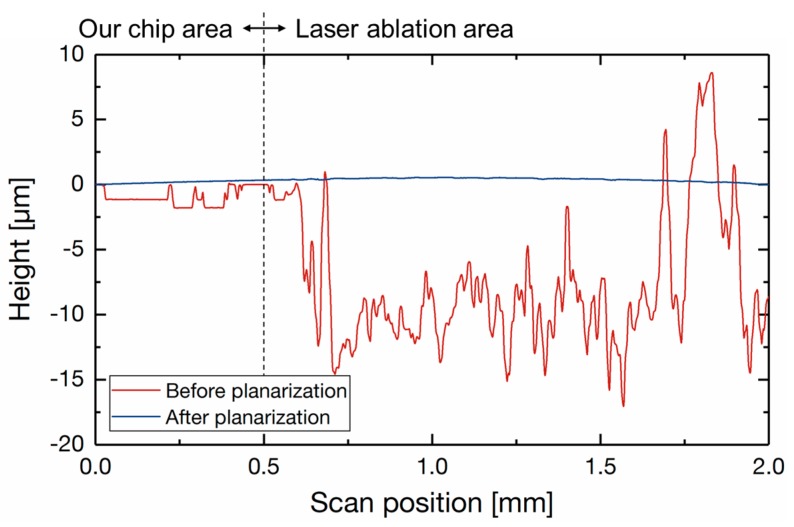
Surface profile of the multi-project wafer (MPW) before and after planarization.

Figure 6 illustrates the fabrication process of the integrated tactile sensor. A multi-project wafer (MPW) fabricated by 0.18 µm CMOS process of Taiwan Semiconductor Manufacturing Co., Ltd. (TSMC) (Hsinchu, Taiwan) was used for the tactile sensor. The MPW has a laser ablation area, as shown in Figure 4a. Figure 7 shows the surface roughness of the MPW. The peak-to-valley roughness of the laser ablation area is over 20 µm, which is not suitable for the subsequent process. In order to decrease the surface roughness, a 20-µm-thick SiO_2_ layer was deposited by plasma enhanced chemical vapor deposition (Figure 6b) and the surface was planarized by chemical mechanical polishing. The CMOS substrate was back-ground to make the surface flat and to reduce the thickness to 300 µm (Figure 6c) [22]. After this planarization process, the surface roughness became small enough for the following processes.

**Figure 8:**
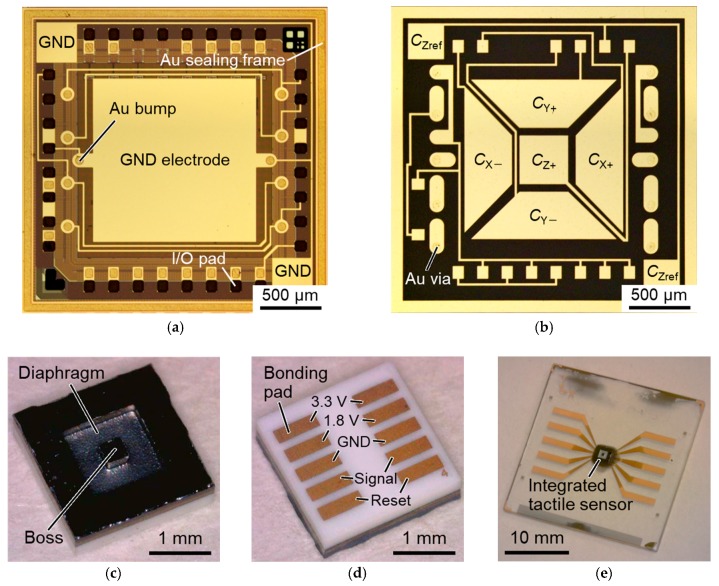
Fabrication results: Optical micrographs of (**a**) CMOS substrate with Au bumps, an Au sealing frame and a GND electrode and (**b**) low temperature cofired ceramic (LTCC) interposer substrate with 3-axis sensing electrodes; (**c**) Front side and (**d**) back side views of a fabricated tactile sensor; (**e**) Surface-mounted tactile sensor on a glass substrate with interconnections.

The SiO_2_ layer was patterned by dry etching for pad opening (Figure 6d). After depositing Au/Pt/Ti seed layers by sputtering, Au of 9 µm thickness was electroplated using a sulfite-based plating solution (Microfab Au 310, Electroplating Engineers of Japan Ltd., Kanagawa, Japan) (Figure 6e). The roles of Au, Pt, and Ti layers are the electrode to electroplate Au, diffusion prevention and adhesion, respectively. The electroplated Au was annealed at 350 °C for 30 min to remove volatile components. After covering the surface of the substrate with a resist for protection, the electroplated Au was planarized by fly-cutting process using a surface planer (DAS8920, DISCO Corporation, Tokyo, Japan) with a diamond bit (Figure 6f) [23]. After removing the resist, the thickness of the electroplated Au was confirmed to be approximately 4.5 µm. The Au/Pt/Ti seed layers were then patterned for forming a ground (GND) electrode and rewiring (Figure 6g). The Au layer was patterned by I_2_/KI solution and Pt/Ti layers were patterned by Ar ion milling. The 3-axis capacitor electrodes were formed on an LTCC interposer (Via-Wafer, Nikko Company, Ishikawa, Japan) by the same procedure as stated above (Figure 6h). Figure 8a,b show the optical micrographs of the CMOS substrate and the LTCC interposer substrate just before bonding, respectively.

The surface of these substrates were activated by Ar ion milling. After alignment by an mask/bonding aligner (MA/BA8, SÜSS MicroTec, Garching, Germany), the substrates were bonded by a wafer bonder (SB6e, SÜSS MicroTec, Garching, Germany) at 300 °C for 30 min with a bonding pressure of about 130 MPa (Figure 6i). A diaphragm was formed in the CMOS substrate by DRIE (Figure 6j). The thickness of the diaphragm was approximately 50 µm. Bonding pads were formed by Au electroplating and patterning the seed layers (Figure 6k). Figure 8c,d show the fabricated sensor after dicing. The sensing diaphragm with the boss is formed on the front side to receive contact force. The bonding pads formed on the backside are for providing the power (3.3 V for analog circuits and input/output (I/O) and 1.8 V for digital circuits), GND, signals, and resets to the CMOS circuit.

The fabricated sensor was surface-mounted on a glass substrate with Au/Ni/Au/Cr interconnections by an anisotropic conductive film (ACF) [24] (Figure 6l). The ACF (MF-301, Hitachi Chemical Co., Ltd., Tokyo, Japan) has Au-plated plastic particles with a diameter of 10 µm and a density of approximately 600 pcs./mm^2^. The bonding pads and the interconnections are electrically connected, keeping the isolation of the adjacent bonding pads. A flip-chip bonder (MODEL-6000, HiSOL, Inc., Tokyo, Japan) was used for surface mounting, and a bonding pressure of 3 N was applied at 170 °C. Figure 8e shows a surface-mounted tactile sensor on a glass substrate with interconnections.

## 4. Experiments and Results

### 4.1. Experimental Method

**Figure 9:**
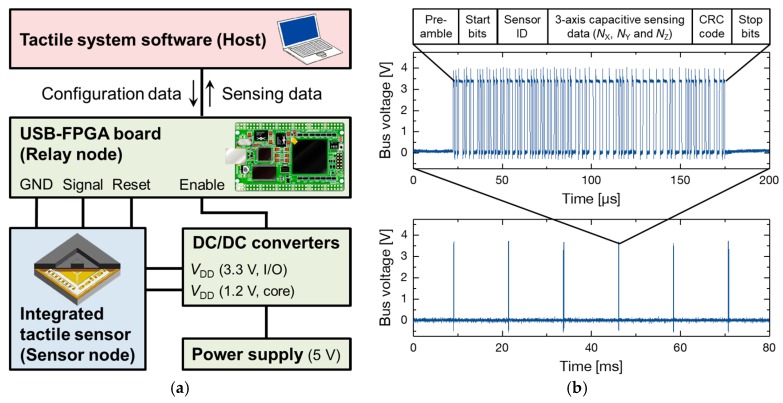
(**a**) Experimental system diagram; (**b**) Digital signal output waveform from the fabricated 3-axis integrated tactile sensor.

Figure 9a shows experimental system diagram. The integrated tactile sensor was connected to a field programmable gate array (FPGA) based relay node (EDA-004, HuMANDATA Ltd., Osaka, Japan) and DC-DC converters. After providing power and configuration data, the tactile sensor recognizes the configuration data via clock and data recovery system [25,26], and starts transmitting sensing data packets. The data packet includes the sensor ID, 3-axis capacitive sensing data, and a cyclic redundancy check (CRC) error check code, as shown in Figure 9b. The clock frequency and data rate of the data packet were 1.34 MHz and 81 samples/s, respectively. The packet data were decoded by the relay node, and the decoded data are analyzed by a host software. The power consumption of the fabricated tactile sensor was estimated as 5.8 mW (1.2 mW for 3.3 V power and 4.6 mW for 1.8 V power).

**Figure 10:**
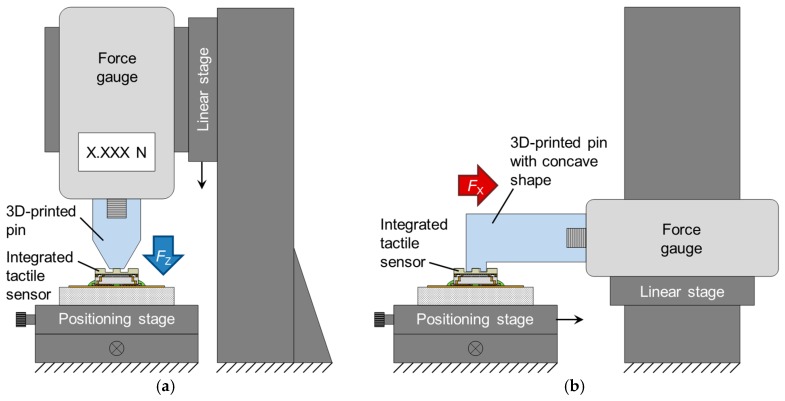
Measurement setup for applying (**a**) normal force and (**b**) shear force.

Figure 10 illustrates the experimental setup. Normal force was applied to the fabricated sensor by pushing a 3D-printed plastic pin vertically using a linear stage via a reference force gauge (ZTS-5N, IMADA Co., Ltd., Aichi, Japan). A 3D-printed pin with a concave shape (200 µm depth) was pushed horizontally to the side surface of the boss by moving a positioning stage to apply shear force.

### 4.2. Results

**Figure 11:**
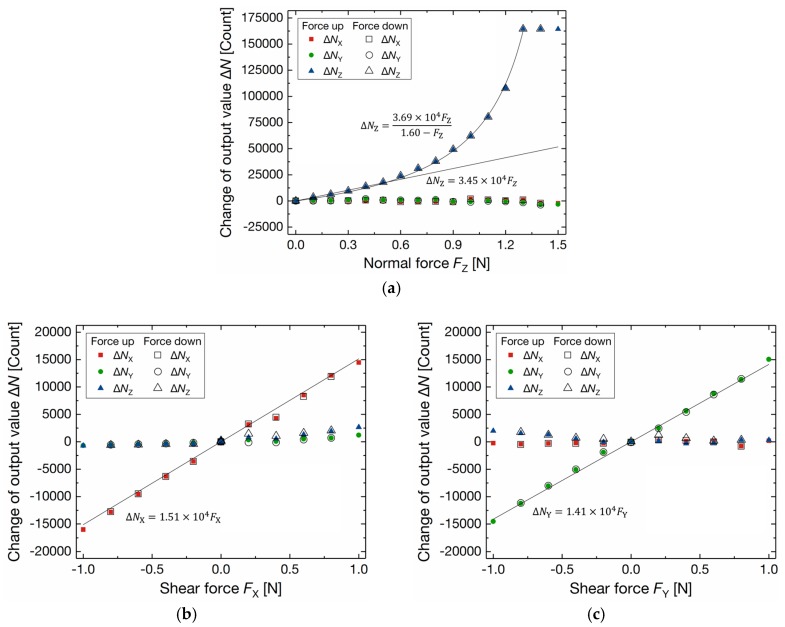
Change of output value of the 3-axis integrated tactile sensor by (**a**) normal force *F*_Z_; (**b**) shear force *F*_X_ and (**c**) shear force *F*_Y_.

Figure 11 plots the change of 3-axis output values for the applied force. Each plot shows the average of 200 output values. As shown in Figure 11a, the *Z*-Axis output value *N*_Z_ increased by applying normal force *F*_Z_ up to 1.3 N, while the change of *N*_X_ and *N*_Y_ are small. The sensitivity for *F*_Z_ up to 0.5 N is calculated as 34.5 Count/mN. The standard deviations of *N*_Z_ at unload condition was 3.5 Count. This corresponds to *F*_Z_ of 0.10 mN, which is calculated by the sensitivity of 34.5 Count/mN. The hysteresis for *F*_Z_ is less than 0.3% full scale (FS). The nonlinear change of *N*_Z_ is due to parasitic capacitance and the nonlinear relationship between the oscillation frequency and the capacitance. Suppose the elastic deformation of the diaphragm, the applied normal force *F*_Z_ is given by

(5) $$F_{Z}=k\Delta d,$$

where *k* and Δ*d* are the stiffness and the deflection of the diaphragm, respectively. The *Z*-Axis sensor capacitance *C*_Z+_ is written as

(6) $$C_{Z+}=\frac{{\varepsilon S}_{Z+}}{d-\Delta d}+C_{p},$$

where ε is permittivity of vacuum, *S*_Z+_ is the area of the *Z*-Axis sensor capacitor *C*_Z+_, *d* is the initial gap of the capacitor electrodes, and *C*_p_ is parasitic capacitance. Combining Equations (1) and (4)–(6) gives the following equation:

(7) $$N_{Z}=f_{\mathrm{Zref}}\cdot \frac{P}{f_{\mathrm{clock}}}-\frac{4.74\times 10^{4}}{\left(\frac{d}{d-\Delta d}C_{Z+,0}+C_{p}\right)+497}\cdot \frac{P}{f_{\mathrm{clock}}},$$

where *C*_Z+,0_ is expressed as *C*_Z+,0_ = ε*S*_Z+_/*d*. The *Z*-Axis output value at unload condition *N*_Z0_ is given by

(8) $$N_{Z0}=f_{\mathrm{Zref}}\cdot \frac{P}{f_{\mathrm{clock}}}-\frac{4.74\times 10^{4}}{C_{Z+,0}+C_{p}+497}\cdot \frac{P}{f_{\mathrm{clock}}}.$$

The change of the *Z*-Axis output value Δ*N*_Z_ (i.e., *N*_Z_ − *N*_Z0_) is written as

(9) $${\Delta N}_{Z}=\frac{4.74\times 10^{4}C_{Z+,0}{\mathrm{PF}}_{Z}}{f_{\mathrm{clock}}{(C}_{p}+{497)(C}_{Z+,0}+C_{p}+497)}\cdot {\left(\frac{C_{Z+,0}+C_{p}+497}{C_{p}+497}\mathrm{kd}-F_{Z}\right)}^{-1},$$

where the units of *f*_clock_, *C*_Z+,0_, and *C*_p_ are MHz, fF, and fF, respectively. Comparing Equation (9) and the equation of the approximate curve in Figure 11a, *k* and *C*_p_ are calculated as 2.9 × 10^5^ N/m and 0.96 pF, respectively. The calculated stiffness is smaller than the simulated one of 3.1 × 10^5^ N/m. The possible reason is the dimension error (e.g., the thickness of the diaphragm was smaller than the designed value). The parasitic capacitance is caused by the CMOS circuit (e.g., pads and I/O buffers) and the interconnections for rewiring. The parasitic capacitance of the CMOS circuit is about several hundred fF. Thicker isolation layer between the CMOS circuit and the interconnections for rewiring enables the decreasing of parasitic capacitance. When large normal force over 1.3 N is applied, *N*_Z_ becomes stable. This is probably because the capacitor electrodes contact by the force.

The *X*-Axis output value *N*_X_ and the *Y*-Axis output value *N*_Y_ were linearly changed by the applied shear force, as shown in Figure 11b,c. The sensitivity is calculated to be 15.1 Count/mN for *X*-Axis and 14.1 Count/mN for *Y*-Axis. The standard deviations of *N*_X_ and *N*_Y_ at unload condition were 12.4 Count and 7.6 Count, respectively. These correspond to *F*_X_ of 0.82 mN and *F*_Y_ of 0.54 mN. The hysteresis is less than 1.7% FS for *X*-Axis and 1.1% FS for *Y*-Axis. The average cross-sensitivity is calculated by the following procedures [27]: (1) the 3-axis output values are converted to measured 3-axis force by the approximation curves shown in Figure 11; and, (2) the average cross-axis sensitivity is calculated as the average of the ratio of the measured cross-axis force to the measured principal-axis force. When *F*_Z_ is applied, the average cross-axis sensitivity is 8.2% for *X*-Axis and 10.4% for *Y*-Axis. In the case that *F*_X_ or *F*_Y_ is applied, the average cross-axis sensitivity is less than 10%. The calculation of the stiffness and the parasitic capacitance for *X*-Axis and *Y*-Axis is not possible by the procedure as stated above because the *Z*-Axis output value is determined by a fixed capacitor (i.e., *f*_Zref_ can be assumed as a constant frequency) and a variable capacitor, while the output values of *X*-Axis and *Y*-Axis are calculated by two variable capacitors. Also, definition of the gap change Δ*d* is difficult for *X*-Axis and *Y*-Axis because the gap change is not uniform due to the tilt of the diaphragm (Figure 3c), although the gap of the *Z*-Axis sensor capacitor changes like a parallel plate type capacitor (Figure 3b). As described in Equations (2)–(4), the output values for each axis is calculated by the difference of the count value of the two capacitors. If the count value of each capacitor can be obtained, estimation of the parasitic capacitances for *X*-Axis and *Y*-Axis will be possible.

**Figure 12:**
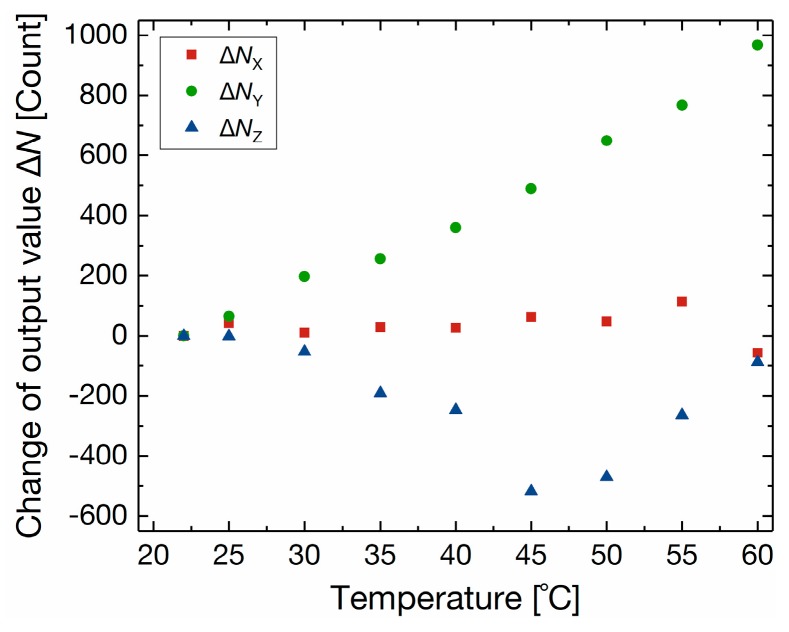
Response of the 3-axis integrated tactile sensor as a function of temperature.

Figure 12 shows the response of the 3-axis integrated tactile sensor to temperature change. The fabricated sensor was heated by a silicone rubber heater from room temperature (22 °C) to 60 °C. The change of the output value corresponds to less than 7.5 mN for *X*-Axis, 69 mN for *Y*-Axis, and 15 mN for *Z*-Axis.

The performance of the fabricated sensor is summarized in Table 1. The performance well fits the tactile sensing for a robot, which we are considering. In addition, the sensor characteristics measured in this study were repeatable because of the almost fatigue-free characteristics of Si and avoidance of the mechanical stress to the analog circuits, which is an important advantage compared with elastomer-based tactile sensors.

## 5. Conclusions

We have developed a 3-axis integrated tactile sensor surface-mountable on a bus line. In order to simplify the fabrication process, a CMOS substrate with circuits for differential capacitive sensing and serial bus communication was flip-bonded on a LTCC interposer substrate by Au-Au thermo-compression bonding, and then the sensing diaphragm was formed from the backside of the CMOS substrate. For the evaluation of the fabricated sensor, normal and shear force was applied independently by 3D-printed pins and movable stages. The fabricated tactile sensor demonstrated 3-axis force detection with a high sensitivity of 15.1 Count/mN for *X*-Axis shear force, 14.1 Count/mN for *Y*-Axis shear force, and over 34.5 Count/mN for *Z*-Axis normal force. The noise at unload condition corresponded to smaller than 1 mN. Also, the hysteresis and the average cross-sensitivity were less than 2% FS and 11%, respectively. The 3-axis integrated tactile sensor developed in this study is useful for the tactile sensation of robots working with human.

## Figures and Tables

**Table 1:** The performance of the fabricated 3-axis integrated tactile sensor.

Force	Sensitivity [Count/mN]	Noise [Count]	Hysteresis [%]	Average Cross-Axis Sensitivity [%]		
				*X*-Axis	*Y*-Axis	*Z*-Axis
*F*_X_ (−1 to 1 N)	15.1	12.4	≤ 1.7	-	5.5	9.0
*F*_Y_ (−1 to 1 N)	14.1	7.6	≤ 1.1	5.2	-	7.2
*F*_Z_ (0 to 1.3 N ^1^)	≥ 34.5	3.5	≤ 0.3	8.2	10.4	-

^1^ *Z*-Axis output value *N*_Z_ saturate when normal force of over 1.3 N is applied.
